# Non-bullous Impetigo: Incidence, Prevalence, and Treatment in the Pediatric Primary Care Setting in Italy

**DOI:** 10.3389/fped.2022.753694

**Published:** 2022-03-31

**Authors:** Elisa Barbieri, Gloria Porcu, Daniele Dona', Nathalie Falsetto, Mirella Biava, Antonio Scamarcia, Luigi Cantarutti, Anna Cantarutti, Carlo Giaquinto

**Affiliations:** ^1^Division of Pediatric Infectious Diseases, Department for Woman and Child Health, University of Padua, Padua, Italy; ^2^Unit of Biostatistics, Epidemiology and Public Health, Department of Statistics and Quantitative Methods, University of Milano-Bicocca, Milan, Italy; ^3^National Centre for Healthcare Research and Pharmacoepidemiology, Department of Statistics and Quantitative Methods, University of Milano-Bicocca, Milan, Italy; ^4^Angelini Pharma S.p.A., Rome, Italy; ^5^Società Servizi Telematici – Pedianet, Padua, Italy

**Keywords:** impetigo, children, primary care, antibiotic therapy, bacterial skin infection

## Abstract

Impetigo is a common skin infection in children. The worldwide prevalence in children is estimated to be 12%, but this may be lower since high-income countries are under-represented. This research aims to evaluate the incidence, prevalence, and management of children with non-bullous impetigo (NBI) residing in Italy. This retrospective cohort study included children up to 14 years of age enrolled in the Pedianet database from January 2004 to June 2018. Events were identified searching ICD9-CM codes (684 and 694.3) and free text fields for a diagnosis of NBI reported during a primary care visit. Diagnoses were manually validated, and events registered within 30-days after the index date were considered follow-ups. Incidence (IR) and prevalence (PR) rates of NBI were stratified by sex, age group, and calendar year. Topical and systemic antibiotic treatments were grouped based on ATC codes. 15,136 NBI episodes occurred in a total cohort of 225,979 children. The overall IR of NBI was 9.5 per 1,000 person-years, and children aged 1–4 years had the highest IR (13.2 per 1,000 person-years). A significant decrease in NBI IR from 13 per 1,000 person-years in 2004 to 7.46 per 1,000 person-years in 2018 (*p* < 0.0001) was noted. Most of the episodes were treated; systemic antibiotics were preferred over topical.

**Conclusion:**

The prevalence of NBI in children in Italy is less than one third than the global estimate and the trend in time is decreasing. Over prescriptions of systemic antibiotics pose a threat to the diffusion of antimicrobial resistance.

## Introduction

Impetigo is one of the most common skin infections (1). It is highly contagious and mostly affects young children and infants, especially preschoolers (2). The global prevalence of impetigo is estimated to be 11.2%, being 2.5-fold higher in children (12.3%) than adults (4.9%) (3). However, because of scarce and outdated studies, prevalence and incidence rates in high-income countries may be overestimated. Indeed, the estimated global prevalence of impetigo is mainly based on children living in tropical, resource-limited situations, with crowded living conditions, poor hygiene, and socio-economic deprivation (1).

There are three types of impetigo: non-bullous, bullous, and ecthyma. The most common form is non-bullous impetigo (NBI), also called *impetigo contagiosa*, accounting for almost 70% of cases (4). The etiology of impetigo varies based on climate and is evolving over time. In temperate climate, it is mainly caused by *Staphylococcus aureus*, while just 5–10% of episodes are caused by *Streptococcus pyogenes* or by a combination of both pathogens (4, 5). Moreover, the pathogenic organism of the bullous form that causes the cleavage within the granular layer of the epidermis is almost always S.aureus (6). Methicillin-resistant S. aureus is detected in some cases of impetigo, ranging from a rate of 1–10% (7–10).

The highly contagious nature of impetigo represents a particular concern in schools and daycare centers (11). To limit the spread of infection, it is recommended that children are kept at home from school or community gathering for 24–48 h after the start of an appropriate antimicrobial therapy, or when all sores have crusted and healed^1^^,^^2^^,^^3^^,^^4^(12). Although symptoms are mild, rare serious complications such as rheumatic heart disease (13) or glomerulonephritis (14) could occur, thus treatment should start promptly.

American and Italian guidelines recommend using topical antibacterial agents for localized impetigo and oral antibiotics for patients with extensive skin lesions unresponsive to topical therapy (15, 16). Antimicrobial resistance (mainly to methicillin and commonly used topical agents, such as mupirocin and fusidic acid) has been increasing over recent years, raising concerns in daily clinical practice (17–20).

Around 50% of patients with impetigo will experience recurrent episodes within 12 months that may require repeated courses of antibiotics, further promoting antibiotic resistance. Risk factors for recurrent NBI have not yet been clarified, although pathogens, host, and environmental factors (including antibiotic misuse) may each play an important role in transmitting resistant bacteria and recurrences (21).

As previously described, non-bullous and bullous impetigo differ in the pathogenesis and etiology. However, most of the studies assessing the burden of impetigo in terms of incidence, prevalence and treatment efficacy have not produced evidence on the two specific forms, leaving open questions on the actual burden of the disease, especially the risk factors and the treatment efficacy.

A new topical quinolone treatment, ozenoxacin, has been developed to tackle the burden of non-bullous impetigo, which seemed to have a low probability of selecting spontaneous resistant mutants in quinolone-susceptible or quinolone-resistant bacterial strains and has shown to be active against MRSA isolates (22).

To better understand the current burden of non-bullous impetigo and the potential value of new treatment for children Italy, it is important to quantify the epidemiology of the disease as well as the risk factors and the treatment efficacy. In this study we estimated the incidence rate, time trend, the prevalence rate and the management of non-bullous impetigo.

## Materials and Methods

### Study Cohort, Data Source, and Case Definition

This retrospective observational cohort study included all children aged from 6 months to 14 years between January 1, 2004, and June 31, 2018, resident in Italy, who were enrolled by one of the 154 family pediatricians (FPs) taking part in the Pedianet network (23) and to whom caregivers had provided consent for the children to be enrolled in the Pedianet Database (24). In Italy, pediatric primary health care within the National Health System is provided free of charge, and each child is enrolled with a FP who is the primary reference point for health-related issues. Pedianet is an organized network of FPs that collects electronic patient records for epidemiological and clinical research purposes. Data generated during routine patient care were collected and handled anonymously, in compliance with Italian regulations, and stored under a unique ID number. Children with missing information about their age or sex in any calendar year during the study period, and children with fewer than two separate medical visits, were excluded from the analyses.

NBI episodes were defined as a visit with a NBI diagnosis identified with ICD9-CM codes (684 and 694.3) or free text (in Italian “impetigine” and related abbreviation) in the diagnosis field. Cases were evaluated manually to exclude any false-positive cases (i.e., bullous impetigo) by a specialist with a clinical background. To avoid duplicates, medical records with the same diagnosis <30 days apart were considered as a follow-up of the initial case.

Recurrent NBI was defined as a child having more than one NBI in the 12 months following the first episode.

Concurrent comorbidities for each NBI episode were identified with ICD9-CM codes or free text in the diagnosis filed and grouped in dermatological, respiratory and others.

This study was conducted according to the principles of the Declaration of Helsinki and the Guidelines of Good Epidemiology Practices. The study design and access to the Pedianet database were approved by the Internal Scientific Committee of So.Se.Pe. Srl, the legal owner of Pedianet.

### Outcomes and Statistical Analysis

The outcomes considered were NBI episodes and antibiotic prescriptions.

Incidence and prevalence rates and their respective 95% confidence intervals (CI) of NBI cases were evaluated. The incidence rate (IR) was calculated by dividing the number of new cases of NBI during the follow-up period by the source population's follow-up period and then further stratified by sex, age group (6–12 months, 1–4 years, 5–14 years) and calendar year (from 2004 to 2018), and expressed as person-years. The Mann-Kendall test was used to determine whether the time series had a monotonic upward or downward trend. Linear regression was then used to determine trend quantification.

Logistic regression models were fitted to estimate the odds ratio (OR) and corresponding 95% CI, measuring the association between exposure to recurrent NBI and the risk of respiratory or dermatological comorbidity episodes. Models were adjusted by sex, age at the start of follow-up, and calendar year. The refernce group was children with simple NBI.

Antibiotic prescriptions (5 ATC digits and 6 ATC digits) were evaluated in a subpopulation of NBI episodes. Children having a concurrent infection (i.e., pharyngitis,otitis media, etc), for which an oral-systemic antibiotic should be prescribed at the time of NBI diagnosis, were excluded from the subpopulation analysis. Children receiving an antibiotic prescription in the 14 days preceding the NBI visits were excluded from the subpopulation analysis because this could have represented an element of bias in issuing the subsequent NBI antibiotic prescription. Stratified analysis for systemic (J01^*^) and topical (D06^*^) antibiotics was performed. The prescribed treatment duration was measured as days of therapy (DOT) and evaluated in prescriptions providing this information. The treatment switch was identified with a second prescription with a different ATC in a time frame of 14 days after the first prescription and defined as an early (days 1–3) or a late (days 4–14) switch. Treatment switch analysis was performed to evaluate the change from a topical to a systemic antibiotic or *vice versa*.

All analyses were performed using the Statistical Analysis System Software (version 9.4; SAS Institute, Cary, NC, USA). Statistical significance was set at the 0.05 level. All *p*-values were two-sided.

## Results

Overall, 13,387 (6%) of children had at least an episode of NBI. Among them, 12,798 were simple NBI (with a number of episodes ranging between 1 (93%) and 5 (0.02%) episodes) compared to 589 recurrent NBI (with a number of episodes ranging between 2 (70%) and 7 (0.17%) episodes). Female children accounted for 46% (5927/12,798) of simple NBI episodes with similar rates among recurrent cases (278/589).

15,136 NBI episodes occurred in a total of 225,979 children. The overall IR of NBI was 9.53 per 1,000 person-years, and children aged 1–4 years had the highest rate (13.24 per 1,000 person-years). A significant decrease in NBI IR was identified in the study period from 13 per 1,000 person-years in 2004 to 7.46 per 1,000 person-years in 2018 (Linear regression *p* trend < 0.0001) (Table 1).

**Table 1:** Non-bullous impetigo cases and incidence rate stratified by sex, age class, and calendar year with corresponding 95% confidence interval. Pedianet 2004–2018.

		**N. cases**	**Person-years**	**Incidence rate** **(CI 95%) x 1,000 person-years**
**Overall**		15,136	1588094.96	9.5 (9.4–9.7)
**Sex**				
	Female	7,010	764035.23	9.17 (8.79–9.40)
	Male	8,126	824059.73	9.86 (9.66–10.08)
**Age group**				
	0–12 months	555	126386.46	4.39 (4.03–4.76)
	1–4 years	5,336	402927.05	13.24 (12.90–13.61)
	5–14 years	9245	1058781.44	8.73 (8.56–8.92)
**Calendar year**				
	2004	848	64758.29	13.09 (12.23–13.98)
	2005	914	78016.49	11.72 (10.97–12.48)
	2006	967	84619.46	11.43 (10.72–12.16)
	2007	935	91270.62	10.24 (9.60–10.91)
	2008	991	98096.87	10.10 (9.49–10.74)
	2009	1,015	103575.24	9.80 (9.21–10.41)
	2010	980	108663.76	9.02 (8.47–9.59)
	2011	1,139	113374.02	10.05 (9.48–10.64)
	2012	1,196	118126.61	10.12 (9.56–10.71)
	2013	1,071	121323.3	8.83 (8.31–9.36)
	2014	1,085	124044.27	8.75 (8.24–9.27)
	2015	1,220	123754.81	9.86 (9.32–10.42)
	2016	1,026	122535.66	8.37 (7.87–8.89)
	2017	880	119459.71	7.37 (6.89–7.86)
	2018	869	116475.84	7.46 (6.97–7.96)

1,727 children with simple NBI (11%) had concurrent comorbidity compared to 15% among recurrent NBI children. Patients with recurrent NBI episodes had twice the risk of having respiratory or dermatological comorbidity compared to patients with non-recurrent NBI (adjusted OR: 2.01 [95%CI: 1.23–3.29] and 2.24 [95%CI: 1.80–2.79]), respectively (Supplementary Table S3 in the Supplementary Material).

**Figure 1:**
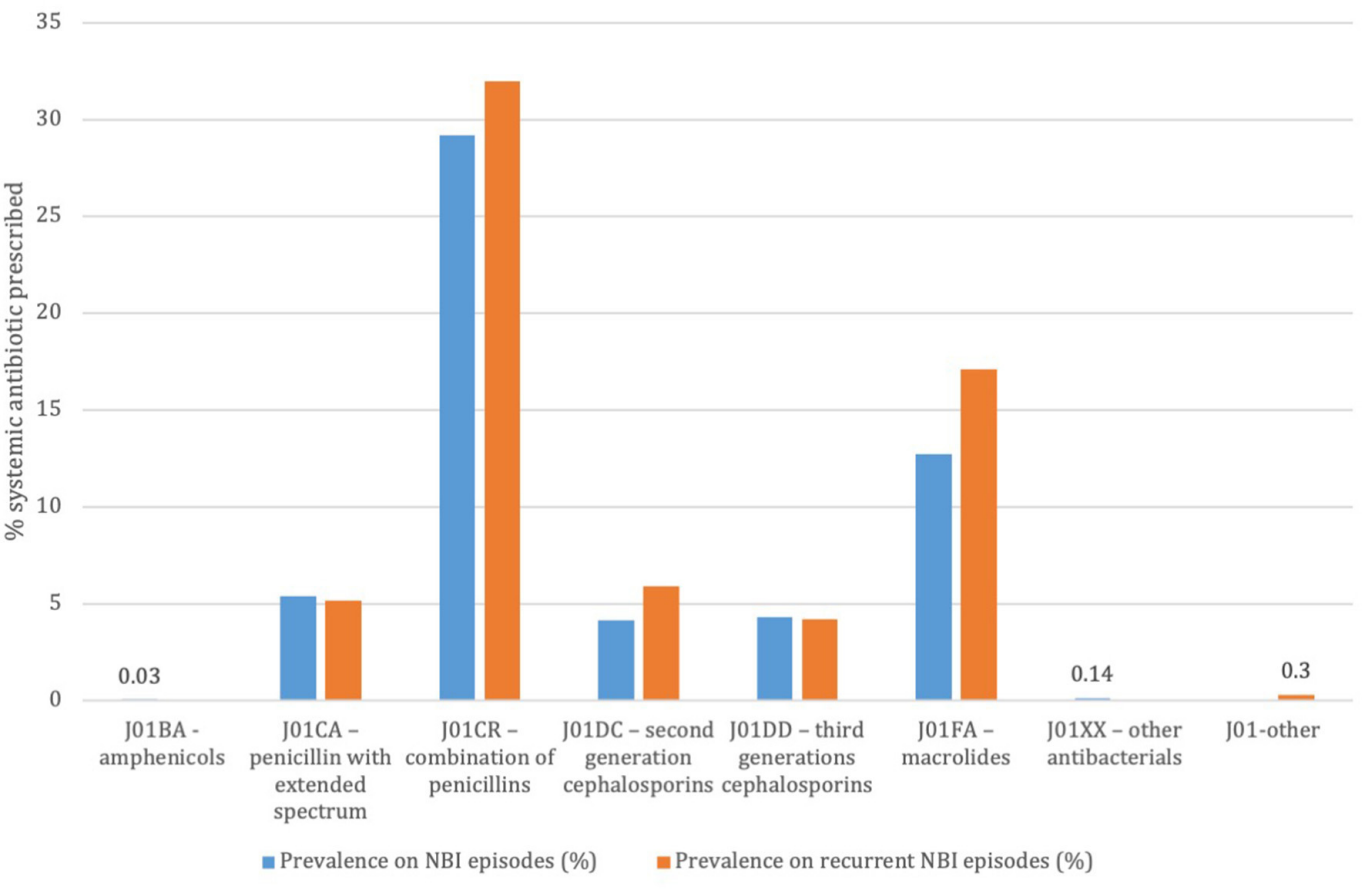
Prevalence of systemic antibiotic prescriptions on total non-bullous impetigo episodes and on recurrent non-bullous impetigo episodes. Pedianet 2004–2018.

Out of 14,569 NBI episodes in children without a concomitant infection or antibiotic prescription in the 14 days preceding the NBI visit 8,297 had a systemic antibiotic prescription. The most prescribed systemic antibiotics were the combination of penicillin with beta-lactams inhibitor (28% and 30%, respectively for simple and recurrent episodes) and macrolides (13 and 16%, respectively) (Figures 1A,B). The mean DOT for the systemic antibiotics was 7.36 (SD 1.99); 34.5% of included therapies were prescribed for seven days, 18.5% for eight days, and 19.5% for 10 days.

**Figure 2:**
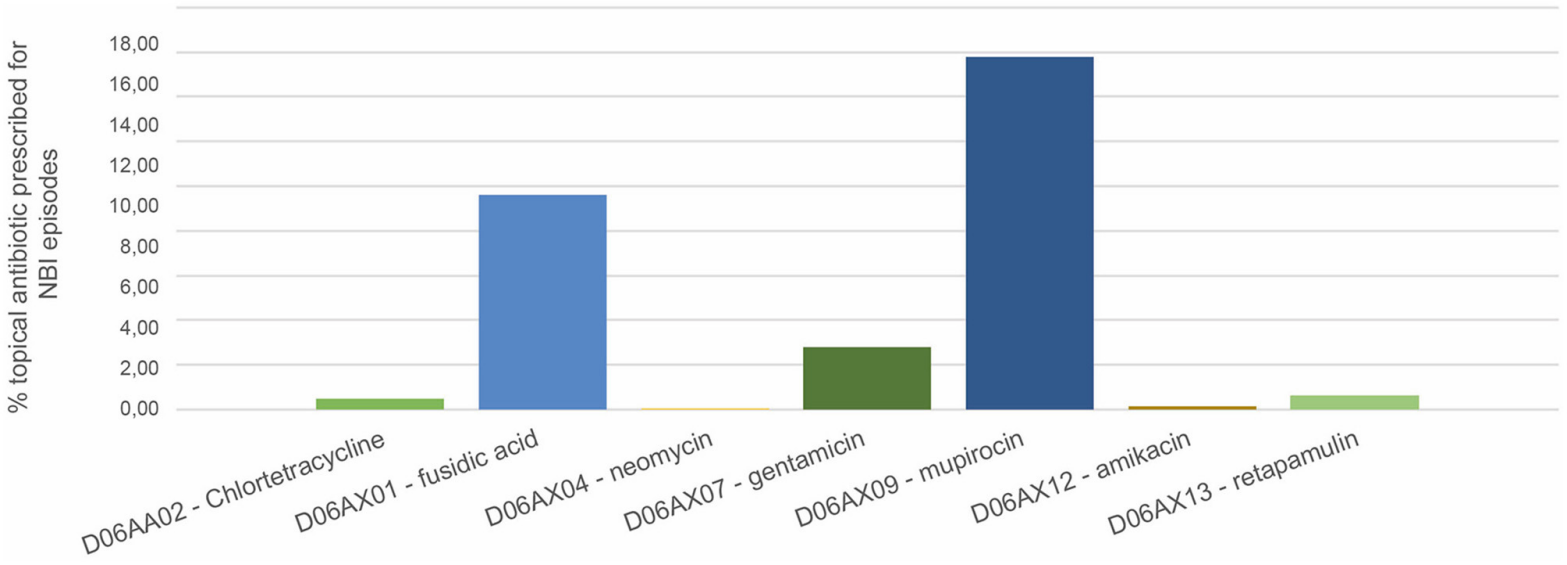
Prevalence of topical antibiotic prescriptions on total non-bullous impetigo episodes. Pedianet 2004–2018.

In total, 4,469 topical antibiotics were prescribed in the subpopulation of 14,569 NBI episodes; in almost half the cases (42.8%), therapy was prescribed for seven days. 18.41% of treatments had ten DOT and 14.77% five DOT. 29.53% of NBI episodes received at least one topical antibiotic; the most prescribed were mupirocin (16%) and fusidic acid (9.6%) (Figure 2).

**Figure 3:**
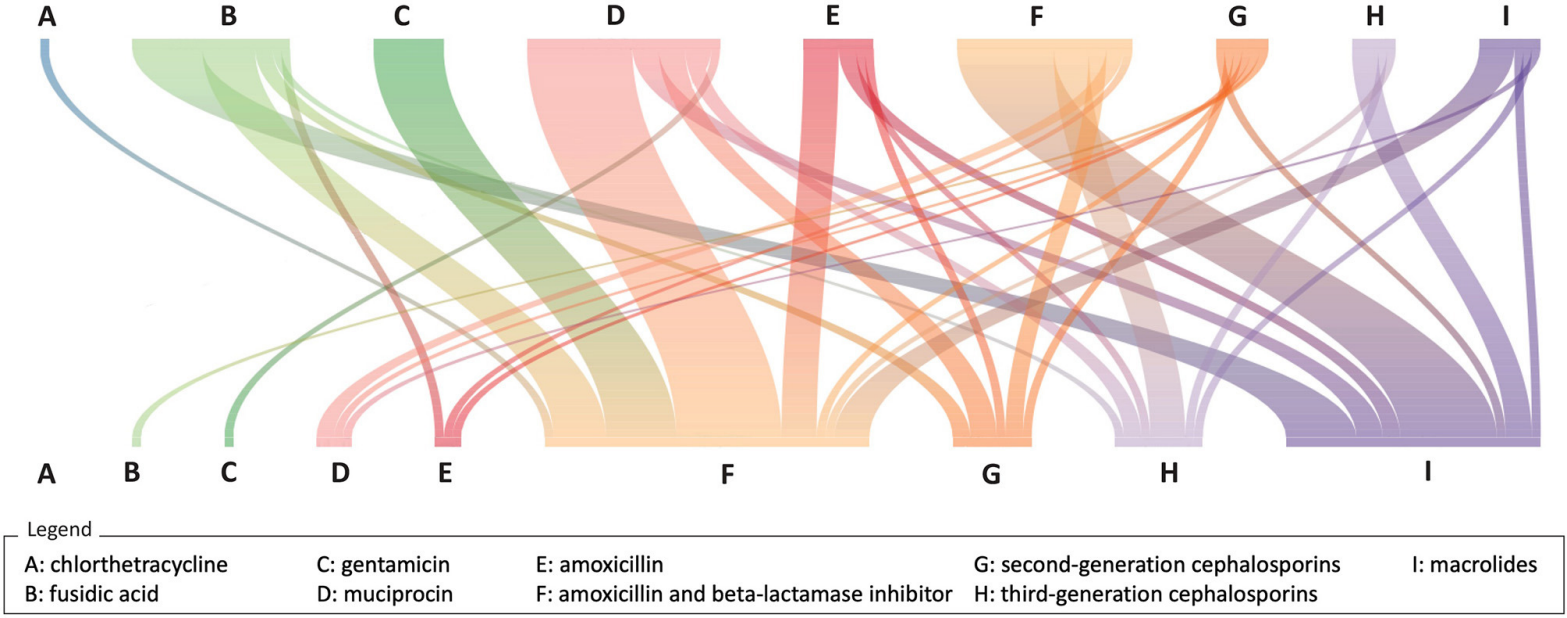
River plot of therapy switches (*N* = 118). The upper row of letters represents the first antibiotic prescription, and the bottom row of letters represents the second antibiotic prescription. The colored lines linking the two rows represent the connection in the same treatment episode. Pedianet, 2004–2018.

Out of 10,148 (67%) NBI episodes with at least an antibiotic prescription, 21 cases had an early and 96 cases a late switch. Therapy switches frequently occurred from topical medication to systemic medication (e.g., from fusidic acid to co-amoxiclav or from mupirocin to co-amoxiclav), involving both early and late switches (Figure 3).

## Discussion

This is the first study assessing the burden of NBI in children residing in Italy. We found that only 6% of children in Italy have at least an episode of NBI, a lower prevalence than that reported in the international literature. Furthermore, our results show a significantly decreasing IR trend over time with the highest value in children 1–4 years old.

The difference with the global estimated prevalence (1, 3) could be explained by the fact that most of the data were from Africa (PR 7%), Asia (PR 7.3%), Oceania (PR 40.2%), Latin America and the Caribbean (PR 15.5%) countries or resource-poor populations in North America (PR 13.3%). Any comparison with other high-income countries is challenging because of the outdated data, the selection of a specific population, and differences in the respective health care systems (1).

The IRs estimated in our study are in line with studies in Northern Europe. In Norway, IR was estimated to be between 9 and 16 per 1,000 person-years from 2001 to 2004 (25), and 3 per 1,000 patient-years in 2012 (26), while a study in the UK estimated impetigo IR to have decreased from 20 to 14 per 1000 person-years from 2004 to 2010 (27). Interestingly, a study conducted in 29 general practices in Utrecht, the Netherlands, estimated the IR of NBI in children to be 64.4 per 1,000 person-years in 2015, with higher cases in summer (29.5%) (28). Different from ours, all the above-mentioned studies considered a broad definition of impetigo, including the bullous form, which seems to be more prevalent in children aged 2–16 years and could reflect a higher IR (27). The reduction in the IRs can be explained by the decline in the impetigo epidemic caused by the epidemic European fusidic acid-resistant impetigo clone. Indeed, in 2003, it was identified a single clone of S. aureus as being the bacterial pathogen involved in the impetigo outbreak in Norway, Sweden, the UK, Ireland, France and the Netherland (26). However, findings from Norway from 2002 to 2012 reported a decline of this fusidic acid-resistant S.aureus that might be correlated to reduced impetigo IRs. In addition to this, different European campaign on the awareness of best infection and prevention control (including hands hygiene) practices have been promoted in the past years, including some special programs targeting to children in schools (29). This, increased infections awareness and might have helped increasing the implementation of best practices, reducing the spread of pathogens causing skin infections.

Respiratory and dermatological comorbidities concurrent with impetigo episodes have already been reported in other studies (28), and the association could be explained considering the etiopathology of the disease. Streptococcal colonization of intact skin precedes the inoculation via skin breaches, and the bacteria could then be transferred from the skin to the upper respiratory tract infection or vice versa (i.e., children with colonization in the nasal mucosae who pick up their nose). This could also explain the higher association found in recurrent NBI cases compared to simple episodes. However, this represent a correlation and not a direct causation since various cofounders to be taken into account.

Most of the NBI episodes were treated either with topical or systemic antibiotics, and treatment switch was rare. According to the Italian intersociety consensus guidelines on the treatment of bacterial skin and soft tissue infection (10), impetigo should be treated with topical antibiotics. Oral antibiotics should only be added to the topical treatments in cases of an extensive disease since inappropriate antibiotic use have proved to increase bacteria resistance (30). Notably, systemic antibiotics were preferred to topical antibiotics (54.8 vs. 29.5%), and macrolides prescriptions were high (16.39%), even if not recommended in the most recent guidelines (16). This might reflect clinicians' fear of a resistant bacterial infection and the possible pressure of parental expectations in receiving antibiotic treatment, as already noted for other conditions (31).

Diffusion of methicillin-resistant *Staphylococcus aureus* (MRSA) strains in the community setting (CA-MRSA) has become a public health concern (32) and in Italy the 35% of *S. aureus* isolates from blood, and cerebrospinal fluid is resistant to methicillin (33). Even if no increasing trend has been recorded over the years, resistance rates in Italy are double the median resistance rate in Europe (34).

Consequently, soft-skin and tissue infections caused by MRSA may be more expensive and difficult to treat (34–36) with relatively fewer antibiotic agents available to treat MRSA infections (32). Moreover, the available agents have substantial limitations, and the development of new antibiotics has slowed over the years (37, 38).

The strength of our study is its size, its generalizability, and its representative coverage of pediatric patients from 2004 to 2018 (24). In this study, 75% of the population referring to a FP enrolled in Pedianet provided their consent. In Italy, it is mandatory to be enrolled in the primary care system, and children are assigned to their FP based on the proximity of their home to the FP ambulatory. Given this, we have no evidence to support a selection bias. Furthermore, patient records are generated during routine patient care using standard software, and data are automatically sent to a centralized system that allows high data reliability.

A limitation lies in the study's retrospective nature. The possibility that at least a few NBI cases were seen in an emergency room without being reported to the FP cannot be excluded. However, such cases would likely have been identified later because a follow-up examination by the FP is nearly always recommended after discharge, especially for younger children. Second, NBI diagnoses were based on clinical evaluation and, even if the dataset was manually validated, the impossibility of confirming clinical assessment is a well-recognized limitation in working with real-world data because it may be subjective to the attending clinician. Third, we assessed antibiotic treatment prescriptions written by the FP, and we cannot exclude that the few cases who had a dermatology visit (0.25% of the total) had an antibiotic prescription written by the specialist. Finally, we assessed the treatment switch, but it was not possible to assess the reason for this.

A possible solution for reducing the spread of antibiotic resistance is decreasing the use of inappropriate antibiotic prescribing, thus diminishing the antibiotic pressure exerted on bacteria. Antimicrobial stewardship programs proved to be a useful tool in reducing bacterial resistance achieved through a more responsible use of antibiotics. Various programs have been implemented over recent years, supporting clinicians in choosing the most appropriate antibiotic regimen, with a few focused on skin and soft tissue infection, especially in the community setting. Before establishing a stewardship program, surveillance information on common bacteria is needed to evaluate the best treatment.

## Conclusion

Our study reported a decrease in non-bullous impetigo incidence rate from 2004 to 2018, with a preference for systemic antibiotic treatment compared to topical treatment that is not in line with current guidelines. The contagious nature of impetigo infection makes the condition of particular concern for the diffusion of antimicrobial resistance, and further surveillance studies assessing the resistance rate to the most common treatments are needed to implement stewardship programs.

## Data Availability Statement

The data analyzed in this study is subject to the following licenses/restrictions: The data used in this study cannot be made available in the article, the Supplementary Material or in a public repository due to Italian data protection laws. The anonymized datasets generated during and/or analyzed during the current study can be provided on reasonable request, from the corresponding author, after written approval by the Internal Scientific Committee. Requests to access these datasets should be directed to Internal Scientific Committee (info@pedianet.it).

## Ethics Statement

Ethical review and approval was not required for the study on human participants in accordance with the local legislation and institutional requirements. Written informed consent from the participants' legal guardian/next of kin was not required to participate in this study in accordance with the national legislation and the institutional requirements.

## Author Contributions

CG, MB, LC, and NF had contributed to the study concept. EB, AC, LC, and CG contributed to the study design and methodology. AS performed the data selection and validation. EB and DD performed the data quality check. GP and AC analyzed the data. EB, AC, AS, and GP had full access to all the data in the study and take responsibility for the integrity of the data and the accuracy of the data analysis. EB, AC, GP, and DD prepared the draft manuscript. AS, LC, MB, and NF contributed to and reviewed the manuscript. EB approved the final manuscript, responsible for the overall content as guarantor, and attests that all listed authors meet authorship criteria. All authors meet the International Committee of Medical Journal Editors (ICMJE) criteria for authorship for this manuscript, take responsibility for the integrity of the work as a whole, and have given final approval for the version to be published. All authors contributed to the article and approved the submitted version.

## Funding

This work was supported by an unrestricted grant of Angelini Pharma S.p.A. (Italy). The funders had no role in the design of the study; in the collection, analyses, or interpretation of data; in the writing of the manuscript, or in the decision to publish the results.

## Author Disclaimer

The opinions expressed are those of the authors with no interference from Angelini Pharma S.p.A.

## Conflict of Interest

NF and MB are employed at Angelini Pharma S.p.A., who is the marketing authorization holder of an ozenoxacin-based topical antibiotic used to treat NBI in Italy (Dubine^®^). The remaining authors declare that the research was conducted in the absence of any commercial or financial relationships that could be construed as a potential conflict of interest.

## Publisher's Note

All claims expressed in this article are solely those of the authors and do not necessarily represent those of their affiliated organizations, or those of the publisher, the editors and the reviewers. Any product that may be evaluated in this article, or claim that may be made by its manufacturer, is not guaranteed or endorsed by the publisher.

## Footnotes

^1^https://deputyprimeminister.gov.mt/en/health-promotion/idpcu/Documents/Impetigo%20fact%20sheet%20for%20schools.pdf (accessed January 27, 2021).

^2^https://www.health.nsw.gov.au/Infectious/factsheets/Pages/impetigo.aspx#:~:text=To%20prevent%20impetigo%20children%20should,scabs%20or%20pick%20their%20nose (accessed January 27, 2021).

^3^https://www.hse.ie/eng/about/who/healthwellbeing/infectcont/sth/info/impetigo-patient-info-leaflet.pdf (accessed January 27, 2021).

^4^https://www.gov.uk/government/publications/health-protection-in-schools-and-other-childcare-facilities/chapter-9-managing-specific-infectious-diseases#impetigo (accessed January 27, 2021).

## Abbreviations

ATC: Anatomical therapeutic chemical
CI: Confidence interval
DOT: Days of therapy
FP: Family pediatricians
ICD-9 CM: International Classification of Diseases, Ninth Revision, Clinical Modification
IR: Incidence rate
MRSA: Methicillin-resistant *Staphylococcus aureus*
NBI: Non-bullous impetigo
PR: Prevalence rate
SD: Standard deviation.
