# Supplementary material for: Non-bullous Impetigo: Incidence, Prevalence, and Treatment in the Pediatric Primary Care Setting in Italy
Source: Front Pediatr. 2022 Mar 31;10:753694. doi: 10.3389/fped.2022.753694 (PMC9008221; doi:10.3389/fped.2022.753694)
Supplement: Supplementary file 1 [file Data_Sheet_1.DOCX]

**Supplementary material**

Figures and Tables

Figure S1. Non-bullous impetigo annual incidence rate (dots) with 95% confidence intervals (whiskers). The dotted line represents the linear trend. Pedianet 2004-2018

Table S2. Prevalence of comorbidities reported in the population with non-bullous impetigo divided by comorbidity and body district. Pedianet 2004-2018

Table S2. Prevalence of comorbidities reported in the population with recurrent non-bullous impetigo divided by comorbidity and body district. Pedianet 2004-2018

Table S3 Odds ratio of having respiratory or dermatological comorbidity in patients with recurrent NBI episodes and patients with non-recurrent NBI reported as crude and adjusted (for sex, age at the start of follow-up, and calendar year) values. Pedianet 2004-2018

Table S4 Early and late therapy switch by couples of antibiotic. Pedianet 2004-2018.

Figure S1 Non-bullous impetigo annual incidence rate (dots) with 95% confidence intervals (whiskers). The dotted line represents the linear trend. Pedianet 2004-2018


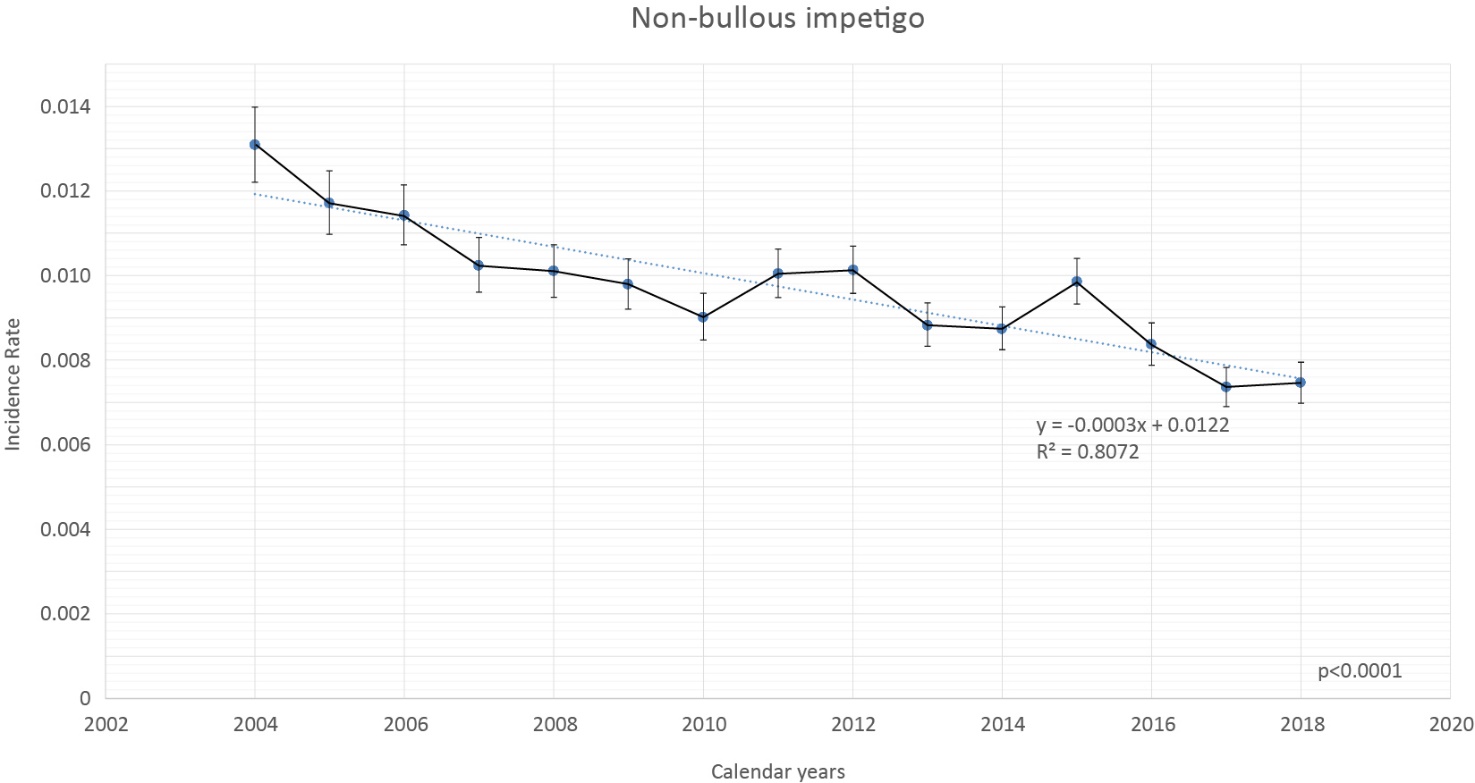


Table S1 Prevalence of comorbidities reported in the population with non-bullous impetigo divided by comorbidity and body district. Pedianet 2004-2018

| Comorbidities | District | N | Prevalence on total NBI (%) |
| --- | --- | --- | --- |
| Rash | Derma | 579 | 3.83 |
| Dermatitis | Derma | 223 | 1.47 |
| Pyoderma | Derma | 201 | 1.33 |
| Wound | Derma | 69 | 0.46 |
| Insect sting | Derma | 65 | 0.43 |
| Eczema | Derma | 58 | 0.38 |
| Herpes | Derma | 55 | 0.36 |
| Hand - foot - mouth syndrome | Derma | 31 | 0.20 |
| Varicella | Derma | 23 | 0.15 |
| Folliculitis | Derma | 10 | 0.07 |
| Prickly heat | Derma | 9 | 0.06 |
| Molluscum contagiosum | Derma | 8 | 0.05 |
| Urticaria | Derma | 8 | 0.05 |
| Prurigo strofulo | Derma | 7 | 0.05 |
| Angular cheilitis | Derma | 5 | 0.03 |
| Burn | Derma | 5 | 0.03 |
| Erysipelas | Derma | 4 | 0.03 |
| Mycosis | Derma | 4 | 0.03 |
| Wart | Derma | 4 | 0.03 |
| Excoriation | Derma | 3 | 0.02 |
| Carbuncles | Derma | 3 | 0.02 |
| Pseudotinea amiantacea | Derma | 3 | 0.02 |
| Scarlet fever | Derma | 3 | 0.02 |
| Tinea capitis | Derma | 3 | 0.02 |
| Cellulite | Derma | 2 | 0.01 |
| Dermatophytosis | Derma | 2 | 0.01 |
| Unspecified viral exanthems | Derma | 2 | 0.01 |
| Fistula | Derma | 2 | 0.01 |
| Whitlow | Derma | 2 | 0.01 |
| Pediculosis | Derma | 2 | 0.01 |
| perionichia | Derma | 2 | 0.01 |
| Sore | Derma | 2 | 0.01 |
| Pityriasis | Derma | 2 | 0.01 |
| Scabies | Derma | 2 | 0.01 |
| Exantema subitum | Derma | 2 | 0.01 |
| Abscess | Derma | 2 | 0.01 |
| Acne | Derma | 1 | 0.01 |
| Condyloma acuminata | Derma | 1 | 0.01 |
| Keloid | Derma | 1 | 0.01 |
| Cradle cap | Derma | 1 | 0.01 |
| Disidrosi | Derma | 1 | 0.01 |
| Cutaneous infection | Derma | 1 | 0.01 |
| Hyperhidrosis | Derma | 1 | 0.01 |
| Lichen striatus | Derma | 1 | 0.01 |
| Onychomycosis | Derma | 1 | 0.01 |
| Pemphigus | Derma | 1 | 0.01 |
| Perionyxis | Derma | 1 | 0.01 |
| Tick bite | Derma | 1 | 0.01 |
| Crosti-Gianotti syndrome | Derma | 1 | 0.01 |
| Cutaneous virosis | Derma | 1 | 0.01 |
| Pharyngitis | Respiratory | 64 | 0.42 |
| Upper respiratory tract Infection (not specified) | Respiratory | 33 | 0.22 |
| Acute otitis media | Respiratory | 32 | 0.21 |
| Rhinitis | Respiratory | 24 | 0.16 |
| Laryngotracheitis | Respiratory | 22 | 0.15 |
| Bronchitis/Bronchitis | Respiratory | 16 | 0.11 |
| Acute nasopharyngitis | Respiratory | 15 | 0.10 |
| Essudative otitis media | Respiratory | 7 | 0.05 |
| Acute sinusitis | Respiratory | 6 | 0.04 |
| Asthma | Respiratory | 3 | 0.02 |
| Conjunctivitis | Other | 17 | 0.11 |
| Gingivostomatitis | Other | 11 | 0.07 |
| Influenza | Other | 7 | 0.05 |
| Whitlow | Other | 5 | 0.03 |
| Candidiasis | Other | 4 | 0.03 |
| Coxachiosi | Other | 4 | 0.03 |
| Balanoposthitis | Other | 3 | 0.02 |
| Lymphadenitis | Other | 3 | 0.02 |
| Flu | Other | 3 | 0.02 |
| Non specified viral infection | Other | 3 | 0.02 |
| Reactive adenite | Other | 2 | 0.01 |
| Fever | Other | 2 | 0.01 |
| Afta | Other | 1 | 0.01 |
| Blepharoconjunctivitis | Other | 1 | 0.01 |
| Chalazion | Other | 1 | 0.01 |
| Colon irritabile | Other | 1 | 0.01 |
| Dacryocystitis | Other | 1 | 0.01 |
| Unspecified disorders of the pituitary and its hypothalamic control | Other | 1 | 0.01 |
| Unspecified gastroenteritis | Other | 1 | 0.01 |
| Chilblain | Other | 1 | 0.01 |
| Gingivitis | Other | 1 | 0.01 |
| Congenital ichthyosis | Other | 1 | 0.01 |
| Acute lymphatic leukemia | Other | 1 | 0.01 |
| Lymphadenomegaly | Other | 1 | 0.01 |
| Oxyuriasis | Other | 1 | 0.01 |
| Fifth disease | Other | 1 | 0.01 |
| Fissures | Other | 1 | 0.01 |
| Ibuprofen adverse event | Other | 1 | 0.01 |
| Bacterial and viral superinfection | Other | 1 | 0.01 |
| Vaginitis | Other | 1 | 0.01 |
| Intestinal and respiratory virosis | Other | 1 | 0.01 |
| **Total** |  | ***1727*** | ***11.41*** |

Table S2. Prevalence of comorbidities reported in the population with recurrent non-bullous impetigo divided by comorbidity and body district. Pedianet 2004-2018

| Comorbidity | District | N | Prevalence on total recurrent NBI |
| --- | --- | --- | --- |
| Rash | Derma | 97 | 6.79 |
| Pyoderma | Derma | 23 | 1.61 |
| Atopic dermatitis | Derma | 17 | 1.19 |
| Dermatitis | Derma | 11 | 0.77 |
| Wound | Derma | 5 | 0.35 |
| Eczema | Derma | 3 | 0.21 |
| Contact dermatitis | Derma | 2 | 0.14 |
| Erysipelas | Derma | 2 | 0.14 |
| Fistula | Derma | 2 | 0.14 |
| Insect sting | Derma | 2 | 0.14 |
| Crusted dermatitis | Derma | 1 | 0.07 |
| Diaper dermatitis | Derma | 1 | 0.07 |
| Eczematous dermatitis | Derma | 1 | 0.07 |
| Superficial wound | Derma | 1 | 0.07 |
| Folliculitis | Derma | 1 | 0.07 |
| Abscess injury in the right leg | Derma | 1 | 0.07 |
| Stropuliform urticaria | Derma | 1 | 0.07 |
| Suppurative perionichia on left index | Derma | 1 | 0.07 |
| Epidemic megaloeritema | Derma | 1 | 0.07 |
| Scabies | Derma | 1 | 0.07 |
| Hand - foot - mouth syndrome (coxsackievirus A16) | Derma | 1 | 0.07 |
| Tinea capitis | Derma | 1 | 0.07 |
| Upper respiratory tract Infection (not specified) | Respiratory | 17 | 1.19 |
| Bronchitis/Bronchiolitis | Respiratory | 3 | 0.21 |
| Asthma | Respiratory | 1 | 0.07 |
| Conjunctivitis | Other | 3 | 0.21 |
| Herpes | Other | 3 | 0.21 |
| Pediculosis | Other | 2 | 0.14 |
| Balanoposthitis | Other | 1 | 0.07 |
| Thrush | Other | 1 | 0.07 |
| Angular cheilitis | Other | 1 | 0.07 |
| Coxachiosi | Other | 1 | 0.07 |
| Herpetic gingivostomatitis | Other | 1 | 0.07 |
| Herpes simplex with other complications | Other | 1 | 0.07 |
| Labial commissure fissures | Other | 1 | 0.07 |
| Stomatitis | Other | 1 | 0.07 |
| Prickly heat | Other | 1 | 0.07 |
| **Total** |  | ***214*** | ***14.99*** |

Table S3 Odds ratio of having respiratory or dermatological comorbidity in patients with recurrent NBI episodes and patients with non-recurrent NBI reported as crude and adjusted (for sex, age at the start of follow-up, and calendar year) values. Pedianet 2004-2018

| **Comorbidity** | **N children** | | **N outcome** | | **OR (95% CI)** | |
| --- | --- | --- | --- | --- | --- | --- |
|  | N recurrent | N simple | N recurrent | N simple | Crude | Adj |
| **Respiratory** | 589 | 12798 | 18 | 198 | 2.01 (1.23 - 3.28) | 2.01 (1.23 - 3.29) |
| **Dermatological** | 589 | 12798 | 109 | 1166 | 2.27 (1.83 - 2.81) | 2.24 (1.80 - 2.79) |

Table S4 Early and late antibiotic therapy switch. Pedianet 2004-2018.

|  | SWITCH (J01 and D06A) | |
| --- | --- | --- |
|  | N | % |
| **EARLY SWITCH** | **21** | **0.17** |
| D06AX09 -J01CR02 | 6 | 28.57 |
| D06AX09 -J01DC04 | 2 | 9.52 |
| J01CR02 -J01FA09 | 2 | 9.52 |
| D06AX01 -J01CR02 | 1 | 4.76 |
| D06AX01 -J01DC04 | 1 | 4.76 |
| D06AX01 -J01FA11 | 1 | 4.76 |
| D06AX07 -J01CA04 | 1 | 4.76 |
| D06AX09 -J01CA04 | 1 | 4.76 |
| D06AX09 -J01FA09 | 1 | 4.76 |
| J01CA04 -J01DC04 | 1 | 4.76 |
| J01CR02 -J01CA04 | 1 | 4.76 |
| J01CR02 -J01FA10 | 1 | 4.76 |
| J01DD08 -J01CR02 | 1 | 4.76 |
| J01DD08 -J01FA09 | 1 | 4.76 |
| J01DD08-J01FA09 | 1 | 4.55 |
| **LATE SWITCH** | **96** | **0.76** |
| D06AX07-J01CR02 | 8 | 8.33 |
| D06AX09-J01CR02 | 8 | 8.33 |
| D06AX01-J01CR02 | 6 | 6.25 |
| J01CR02-J01FA09 | 6 | 6.25 |
| J01CR02-J01FA10 | 5 | 5.21 |
| D06AX01-J01FA09 | 4 | 4.17 |
| D06AX09-J01CR02 | 4 | 4.17 |
| J01CA04-J01CR02 | 4 | 4.17 |
| D06AX01-J01FA11 | 3 | 3.13 |
| D06AX09-J01FA09 | 3 | 3.13 |
| J01DD08-J01FA09 | 3 | 3.13 |
| D06AX09-J01DC04 | 2 | 2.08 |
| D06AX09-J01DD08 | 2 | 2.08 |
| J01CR02-D06AX09 | 2 | 2.08 |
| J01CR02-J01DD13 | 2 | 2.08 |
| J01FA09-J01CR02 | 2 | 2.08 |
| D06AA02-J01CR02 | 1 | 1.04 |
| D06AX01-J01CA04 | 1 | 1.04 |
| D06AX01-J01DC02 | 1 | 1.04 |
| D06AX01-J01DC04 | 1 | 1.04 |
| D06AX01-J01DD13 | 1 | 1.04 |
| D06AX01-J01FA10 | 1 | 1.04 |
| D06AX09-D01BA02 | 1 | 1.04 |
| D06AX09-D06AX07 | 1 | 1.04 |
| D06AX09-J01DC10 | 1 | 1.04 |
| J01CA04-J01DC04 | 1 | 1.04 |
| J01CA04-J01DD13 | 1 | 1.04 |
| J01CA04-J01FA09 | 1 | 1.04 |
| J01CA04-J01FA10 | 1 | 1.04 |
| J01CR02-J01CA04 | 1 | 1.04 |
| J01CR02-J01DC02 | 1 | 1.04 |
| J01CR02-J01DC10 | 1 | 1.04 |
| J01CR02-J01DD04 | 1 | 1.04 |
| J01CR02-J01DD08 | 1 | 1.04 |
| J01DC02-J01FA09 | 1 | 1.04 |
| J01DC04-D06AX01 | 1 | 1.04 |
| J01DC04-D06AX09 | 1 | 1.04 |
| J01DC04-J01CA04 | 1 | 1.04 |
| J01DC04-J01CR02 | 1 | 1.04 |
| J01DC04-J01DC02 | 1 | 1.04 |
| J01DD04-J01DD08 | 1 | 1.04 |
| J01DD08-J01CR02 | 1 | 1.04 |
| J01FA09-D06AX09 | 1 | 1.04 |
| J01FA09-J01DD08 | 1 | 1.04 |
| J01FA10-J01CR02 | 1 | 1.04 |
| J01FA10-J01FA01 | 1 | 1.04 |
| J01FA12-J01CR02 | 1 | 1.04 |
| **Total switch** | **116** | **0.93** |
| **Total J01 and D06A prescriptions** | **12648** |  |
